# Oily fish reduces the risk of acne by lowering fasting insulin levels: A Mendelian randomization study

**DOI:** 10.1002/fsn3.4054

**Published:** 2024-03-06

**Authors:** Ting Cheng, Dongdong Yu, Bingqing Liu, Xingying Qiu, Qi Tang, Geng Li, Li Zhou, Zehuai Wen

**Affiliations:** ^1^ Second Clinical College of Guangzhou University of Chinese Medicine Guangzhou China; ^2^ First Affiliated Hospital of Anhui University of Chinese Medicine Hefei China; ^3^ Guangdong Provincial Hospital of Chinese Medicine (Second Affiliated Hospital of Guangzhou University of Chinese Medicine) Guangdong Provincial Academy of Chinese Medical Sciences Guangzhou China; ^4^ Science and Technology Innovation Center of Guangzhou University of Chinese Medicine Guangzhou China

**Keywords:** acne, fasting insulin, Mendelian randomization analysis, oily fish intake, risk factor

## Abstract

Meat intake, particularly from oily fish, has been associated with various chronic diseases. However, its relationship with acne has always been controversial. Therefore, we have adopted Mendelian randomization (MR) analysis to investigate the causal relationship between different types of meat intake and acne. The exposure and outcome datasets for this study were obtained from the Integrative Epidemiology Unit (IEU) Open GWAS project. Seven datasets on meat intake were included, which consisted of non‐oily fish, oily fish, lamb/mutton, poultry, pork, beef, and processed meat. The main methods used for MR analysis were inverse variance weighted, weighted median, and MR‐egger. To ensure the accuracy of the results, heterogeneity, pleiotropy, and Mendelian randomization pleiotropy residual sum and outlier (MR‐PRESSO) analyses were conducted. Additionally, an analysis of four risk factors (fasting insulin, insulin resistance, total testosterone level, and estradiol level) was performed to investigate the underlying mechanisms linking statistically significant meat intake to acne. Oily fish intake was found to be a protective factor for acne (OR: 0.22, 95% CI: 0.10–0.49, *p* < .001), and it was also observed that oily fish intake can reduce the level of fasting insulin by the IVW method (OR: 0.89, 95% CI: 0.81–0.98, *p* = .02). No causal relationship was identified between other types of meat intake and acne. The intake of oily fish reduces the risk of acne by lowering fasting insulin levels.

## Graphical abstract

We have adopted Mendelian randomization (MR) to investigate the causal relationship between different types of meat intake (processed meat, poultry, beef, non‐oily fish, oily fish, pork, and lamb/mutton) and acne. We found that the intake of oily fish reduces the risk of acne by lowering fasting insulin levels.

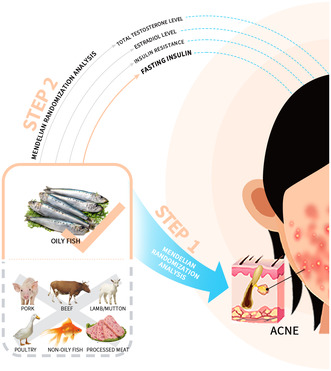

## 1 INTRODUCTION

Acne is a highly prevalent dermatological condition that affects millions of individuals globally, particularly young adults. It is considered one of the most common skin disorders, with a lifetime incidence approaching 100% (Collier et al., 2008; Thiboutot, 2008). The pathogenesis of acne involves four primary mechanisms, including the colonization of *Cutibacterium acnes* (once known as *Propionibacterium acnes*) (Dekio et al., 2019), follicular hyperkeratinization, heightened sebum production, and inflammation (Dessinioti, 2022). These mechanisms are believed to contribute to the development of the disease, along with interactions involving factors such as genetic susceptibility, medication use, diet, occupation, pollution, climate, lifestyle, hormonal influences, and the immune system (Cong et al., 2019; Dréno et al., 2018; Gollnick, 2015; Kircik, 2019). For many years, there has been a commonly held belief among the general public that dietary habits play a substantial role in the occurrence, duration, and changes in the condition of acne. Nonetheless, the current understanding of the relationship between diet and acne is still uncertain and remains a topic of ongoing debate. As an illustration, the American Academy of Dermatology stated in 2007 that restricting calorie intake does not provide any benefits for treating acne. Furthermore, there is a lack of substantial evidence to substantiate the idea that the consumption of specific “food enemies” can directly cause acne (Strauss et al., 2007). Conversely, research conducted from 2009 to 2020 verifies that acne can be caused by high glycemic index (GI) foods, dairy products, high‐fat foods, and chocolate, while the consumption of fatty acids, fruits, and vegetables can help prevent acne. This proves to be consistent due to research with the Inuit population (from northern Canada) that no cases of acne were found when they ate a traditional diet composed of marine and terrestrial mammals (e.g., seal and caribou), as well as wild birds and fish (Dall'Oglio et al., 2021; Kenny et al., 2018).

Although the relationship between meat consumption and acne has received significant attention, there is currently limited research directly focusing on the association between meat intake and acne. On the one hand, many existing studies heavily rely on retrospective observational study designs, which are prone to recall bias and difficulties in data collection. On the other hand, it is challenging to account for confounding variables such as genetics, hormone levels, and environmental influences in the research process, leading to inconsistent findings. Moreover, despite some studies suggesting a potential link between meat intake and acne development, the specific underlying mechanisms remain unclear. Mendelian randomization (MR) is a statistical technique that utilizes genetic variations as instrumental variables (IVs) to assess the potential causal relationship between exposure factors and outcomes. The utilization of this approach can mitigate the impact of confounding variables and yield more dependable findings in elucidating the correlation between meat consumption and acne. Additionally, this research seeks to identify mediating elements that shape the influence of meat intake on acne, thus enhancing our understanding of the fundamental mechanisms involved in the occurrence and development of acne. These findings not only provide significant indications for further studies but also offer substantial evidence for implementing dietary management strategies among individuals afflicted by acne.

## 2 METHODS

### 2.1 MR assumptions

**FIGURE 1:**
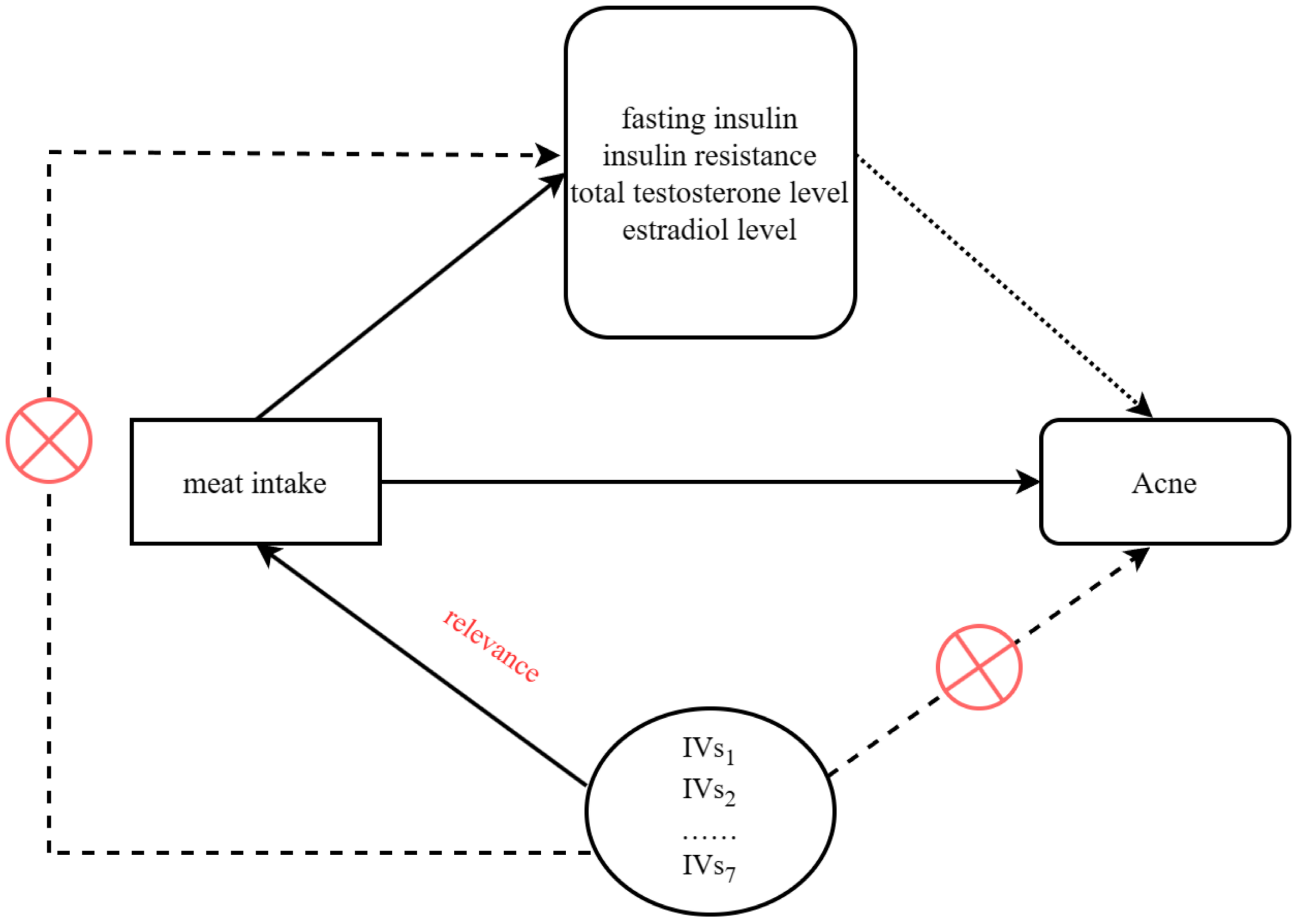
The study design used in this Mendelian randomization (MR) analysis.

Our research must adhere to the three fundamental assumptions of MR analysis, which are relevance, independence, and exclusion restriction: (1) Genetic variants used as IVs are strongly associated with the exposure or risk factor under investigation; (2) IVs do not have any direct effect on the outcome, except through their association with the exposure or risk factor; and (3) IVs are independent of confounding factors that may introduce bias in the observed associations between the exposure or risk factor and the outcome. The design diagram for this MR analysis can be referenced in Figure 1 of the study.

### 2.2 Data sources

Table S1 presents the details of the genome‐wide association study (GWAS) included in the MR analysis. This analysis encompassed seven distinct categories of meat consumption, which comprised non‐oily fish, oily fish, lamb/mutton, poultry, pork, beef, and processed meat. The categorization was supported by the MRC Integrative Epidemiology Unit (MRC‐IEU) at the University of Bristol. The GWAS data for acne were obtained from the FinnGen biological database through the IEU Open GWAS project, including 1299 cases and 211,139 control cases, all of European descent. Additionally, summary‐level GWAS data for fasting insulin were extracted from the Meta‐Analyses of Glucose and Insulin‐related Traits Consortium (MAGIC). The data on insulin resistance, total testosterone levels, and estradiol levels were obtained from a distinct meta‐analysis of GWAS data. Importantly, the populations involved in the exposures and outcomes were distinct, as these datasets were sourced from different consortia. All the GWAS datasets utilized in this MR analysis were sourced from the Integrative Epidemiology Unit (IEU) Open GWAS project (https://gwas.mrcieu.ac.uk/). To mitigate potential biases stemming from population heterogeneity, these datasets exclusively included individuals of European descent. Ethical approval or informed consent was not necessary for the secondary analysis as it was performed using publicly accessible data.

### 2.3 The selection of IVs

The IVs selected for the exposure in this study were chosen based on specific criteria to ensure their validity. These criteria include a strong association with the exposure (*p* < 5e‐8), a low level of linkage disequilibrium (*r* ^2^ < .001), a clumping window of 10,000 kb, and an *F* statistic greater than 10. Additionally, in order to minimize potential alignment uncertainties, we excluded palindromic single‐nucleotide polymorphisms (SNPs) from the analysis, considering their presence in both the exposure and outcome GWASs. Furthermore, the website (http://www.phenoscanner.medschl.cam.ac.uk/) was utilized to exclude SNPs that were associated with the exposure as well as potential confounding variables. To ensure a robust association between IVs and exposure, we calculated the *F* statistic, we calculate (Burgess et al., 2016):

$$\begin{array}{c} F=\left(N-2\right)\times R^{2}\;/\;\left(1-R^{2}\right),R^{2}=\beta^{2}\left(1-\mathrm{EAF}\right)\times 2\mathrm{EAF}, \end{array}$$

*β* ^2^: genetic effect of each IV in exposure, EAF: effect allele frequency, and *N*: sample size. SNPs that satisfy all the aforementioned criteria were utilized in the final MR analysis.

### 2.4 Statistical analysis

The main estimates in our MR analysis were obtained using the inverse‐variance weighted (IVW) method, assuming the validity of all IVs. In more extensive scenarios, we also employed the MR‐Egger method and the weighted median method. A sensitivity analysis was conducted to identify potential pleiotropy and heterogeneity in our MR study. To assess potential horizontal pleiotropy, Cochran's *Q* test was performed using the IVW approach (*p* < .05). The MR‐Egger intercept test was used to detect directional pleiotropy (*p* < .05). In cases where heterogeneity was detected (*p* < .05), the random effects IVW method was utilized to estimate the causal effect. To evaluate and address horizontal pleiotropy, we employed the Mendelian randomization pleiotropy residual sum and outlier (MR‐PRESSO) method. Furthermore, the MR‐PRESSO method allowed us to address level pleiotropy by identifying and removing outliers and assessing the statistical significance of the difference in causal estimates before and after outlier correction (Ong & MacGregor, 2019). The final results of the MR analysis, after the removal of outliers, are presented. Leave‐one‐out analysis is a method in which each genetic variant is systematically removed one at a time to assess its impact on the causal relationship. Visualizations were created using scatter plots, forest plots, and funnel plots.

The MR analysis was performed using the two‐sample MR package (version 0.5.7) and the MR‐PRESSO package (version 1.0) in R software (version 4.2.2). Statistical significance was determined using a threshold of *p* < .05.

## 3 RESULTS

### 3.1 Causal effect from meat intake to acne

In this study, we examined the potential causal relationship between meat intake and acne by considering seven specific dietary factors. The analysis included a range of SNPs, varying from 7 to 61 (Supplementary data), all of which met the criterion of having *F*‐statistics greater than 10 (with the lowest being 14.7). This indicates a strong association with the exposures (Table S2). No evidence of heterogeneity was observed across all seven exposures (as determined by Cochran's *Q* test, *p* > .05), and the MR‐Egger intercept results indicated the absence of directional pleiotropy (*p* > .05) (Table 1). These findings underscore the reliability of our results. The MR‐PRESSO analysis did not identify any outliers in relation to acne for various types of meat (Table S3). Detailed scatter plots, forest plots, leave‐one‐out plots, and funnel plots can be found in Figures S1–S7.

**TABLE 1:** The causal effect of 7 types of meat intake on acne in two sample MR.

Exposures	SNP	Methods	OR (95% CI)	*p*	Test of heterogeneity		Intercept term		
					Cochran's *Q*	*p*	Egger intercept	SE	*p*
Processed meat intake	23	MR Egger	1.59 (0.00–1020.39)	.89	13.81	0.88	<0.001	0.05	.94
Processed meat intake	23	IVW	1.22 (0.34–4.43)	.76	13.81	0.91			
Processed meat intake	23	Weighted median	0.78 (0.13–4.62)	.79					
Poultry intake	7	MR Egger	1.59E+21 (0–1.36E+60)	.33	1.77	0.88	0.56	0.50	.31
Poultry intake	7	IVW	0.08 (0.00–1.51)	.09	3.03	0.81			
Poultry intake	7	Weighted median	0.04 (0.00–1.87)	.10					
Beef intake	14	MR Egger	7.91 (0.00–1.08E+6)	.74	9.01	0.70	0.03	0.08	.72
Beef intake	14	IVW	0.87 (0.12–6.13)	.89	9.15	0.76			
Beef intake	14	Weighted median	0.93 (0.06–13.31)	.96					
Non‐oily fish intake	11	MR Egger	0.00 (0.00–191.93)	.31	10.83	0.29	0.08	0.07	.30
Non‐oily fish intake	11	IVW	0.88 (0.07–10.71)	.92	12.26	0.27			
Non‐oily fish intake	11	Weighted median	0.17 (0.01–4.24)	.28					
Oily fish intake	61	MR Egger	0.05 (0.00–1.56)	.09	56.85	0.56	0.02	0.03	.39
Oily fish intake	61	IVW	0.22 (0.10–0.49)	**<.001**	57.60	0.56			
Oily fish intake	61	Weighted median	0.19 (0.06–0.61)	**.01**					
Pork intake	13	MR Egger	74715.86 (0.02–2.34E+11)	.17	5.72	0.89	−0.10	0.08	.21
Pork intake	13	IVW	3.39 (0.31–37.37)	.32	7.48	0.82			
Pork intake	13	Weighted median	8.47 (0.33–214.81)	.20					
Lamb/mutton intake	31	MR Egger	0.99 (0.00–820.69)	1.00	22.02	0.82	0.01	0.04	.89
Lamb/mutton intake	31	IVW	1.56 (0.32–7.55)	.58	22.04	0.85			
Lamb/mutton intake	31	Weighted median	1.71 (0.19–15.29)	.63					

*Note*: Bold indicates that *p* < .05.

Abbreviations: CI, Confidence interval; IVW, inverse‐variance weighted; MR, Mendelian randomization; OR, Odds ratio; SNP, single nucleotide polymorphisms.

The primary results of the main MR analyses for the seven types of meat intake on acne are presented in Table 1. Oily fish intake (OR: 0.22, 95% CI: 0.10–0.49, *p* < .001) was identified as a protective factor by using the IVW method, and this finding was further confirmed by the weighted median method (OR: 0.19, 95% CI: 0.06–0.61, *p* = .01). The study also revealed that the intake of processed meat (OR: 1.22, 95% CI: 0.34–4.43, *p* = .76), poultry (OR: 0.08, 95% CI: 0.00–1.51, *p* = .09), beef (OR: 0.87, 95% CI: 0.12–6.13, *p* = .89), non‐oily fish (OR: 0.88, 95% CI: 0.07–10.71, *p* = .92), pork (OR: 3.39, 95% CI: 0.31–37.37, *p* = .32), and lamb/mutton (OR: 1.56, 95% CI: 0.32–7.55, *p* = .58) were not associated with acne (all *p* > .05).

### 3.2 Causal effect of oily fish intake on potential acne risk factors

To investigate whether oily fish intake is linked to acne through pleiotropic pathways, we examined its relationship with four acne risk factors: fasting insulin, insulin resistance, total testosterone levels, and estradiol levels. The IVW method, MR‐Egger method, and weighted median method were used for analysis. As shown in Table 2, oily fish intake can reduce the level of fasting insulin via the IVW method (OR: 0.89, 95% CI: 0.81–0.98, *p* = .02). Additionally, no associations were observed between oily fish intake and insulin resistance, total testosterone levels, and estradiol levels (all *p* > .05). The MR‐PRESSO analysis identified outliers associated with oily fish intake and four risk factors. Nevertheless, there was no indication of directional pleiotropy (*p* > .05) (Table S4). Detailed scatter plots, forest plots, leave‐one‐out plots, and funnel plots can be found in Figures S8–S11.

**TABLE 2:** The MR analysis of the associations between oily fish intake and four common risk factors.

Outcome	SNP	Methods	OR (95% CI)	*p*
Fasting insulin	12	MR Egger	1.14 (0.80–1.62)	.49
Fasting insulin	12	IVW	0.89 (0.81–0.98)	**.02**
Fasting insulin	12	Weighted median	0.95 (0.83–1.08)	.40
Insulin resistance	45	MR Egger	0.77 (0.49–1.19)	.24
Insulin resistance	45	IVW	0.99 (0.90–1.09)	.83
Insulin resistance	45	Weighted median	0.96 (0.84–1.10)	.56
Total testosterone levels	58	MR Egger	1.16 (0.85–1.60)	.36
Total testosterone levels	58	IVW	1.07 (0.99–1.16)	.07
Total testosterone levels	58	Weighted median	1.10 (1.00–1.22)	.06
Estradiol levels	60	MR Egger	0.99 (0.92–1.07)	.80
Estradiol levels	60	IVW	1.00 (0.98–1.02)	.85
Estradiol levels	60	Weighted median	1.01 (0.98–1.04)	.47

*Note*: Bold indicates that *p* < .05.

Abbreviations: CI, Confidence interval; IVW, inverse‐variance weighted; MR, Mendelian randomization; OR, Odds ratio; SNP, single nucleotide polymorphisms.

## 4 DISCUSSION

Our research results indicate a significant increase in the risk of acne among individuals who did not consume oily fish compared to those who did. However, no significant associations were found between other types of meat intake and acne. These findings suggest that the effects of different meat types on acne may vary. Additionally, we found that oily fish intake was associated with reduced fasting insulin levels in individuals with acne, but no significant effects were observed on the well‐established risk factors for acne, including insulin resistance, total testosterone levels, and estradiol levels. Importantly, to the best of our knowledge, this is the first MR study to examine the causal relationship between seven distinct meat intake factors and acne while also identifying potential mediating effects.

The consumption of meat has been progressively rising, leading to heightened scrutiny of its influence on health, particularly skin health (Xie et al., 2022). Although meat is renowned for its protein content and nutritional value, its association with acne development continues to spark debate. Early observational studies have put forth a potential connection between red and processed meats and the incidence of acne (National Guideline Alliance (UK), 2021). This is due to their composition and potential effects on inflammation and hormonal balance. Additionally, some studies have indicated that fish, which is rich in omega‐3 fatty acids, may have a positive impact on acne (Di Landro et al., 2012; Jung et al., 2010). These benefits include regulating sebum production, decreasing inflammatory responses, and promoting skin cell turnover. Interestingly, this study utilizes an MR analysis method to confirm the impact of different types of meat intake on acne and reveals that only the consumption of oily fish has a protective effect on acne. Nevertheless, it is crucial to acknowledge that the impact of meat consumption on acne may vary among individuals due to genetic variations, overall dietary patterns, and lifestyle choices. What is more, the specific cooking methods used, such as grilling or frying, as well as the accompanying ingredients, can also influence how meat affects acne symptoms. In summary, individuals concerned about the influence of meat on their acne should seek guidance from healthcare professionals or registered dietitians for personalized dietary recommendations and comprehensive acne management strategies.

Oily fish is widely acknowledged for its abundance of monounsaturated fatty acids (MUFAs) and polyunsaturated fatty acids (PUFAs), docosahexaenoic acid (DHA), vitamin D, high protein content, and other nutrients, especially omega‐3 polyunsaturated fatty acids (ω‐3 PUFAs) and eicosapentaenoic acid (EPA) (Teisen et al., 2020). Examples of oily fish include salmon, sardines, trout, Pacific saury, Spanish mackerel, fresh tuna, and herring (Kim et al., 2019). Extensive research conducted in the past has extensively examined the relationship between the intake of oily fish, its fatty acid composition, and various diseases or conditions, including nonalcoholic fatty liver disease (Tan & Shin, 2022), frailty (Del Brutto et al., 2020), cognitive performance (Del Brutto, Mera, Gillman, Zambrano, & Ha, 2016), cardiovascular disease (Mozaffarian & Wu, 2011), blood pressure levels (Del Brutto, Mera, Gillman, Castillo, et al., 2016), diffuse subcortical damage of vascular origin (Del Brutto et al., 2022), and diabetic retinopathy (Mayor, 2016). In addition, a study has demonstrated that regular consumption of more than six servings of oily fish per week reduces the risk of all‐cause mortality in middle‐aged and older adults with Amerindian ancestry (Del Brutto et al., 2023). Another study has indicated that incorporating oily fish into the diet during pregnancy can alter neonatal immune responses (Noakes et al., 2012). These effects are primarily mediated by the anti‐inflammatory, anti‐oxidative, anti‐arrhythmic, anti‐hypertensive, and anti‐thrombotic properties of long‐chain ω‐3 PUFAs derived from marine sources (Mori, 2014; van Bussel et al., 2011). Some studies have confirmed that oily fatty acids have the ability to kill or inhibit the growth of bacteria (Desbois & Smith, 2010; Kapoor et al., 2021). Seventeen fatty acids (FAs), including myristoleic acid, inhibited *Cutibacterium acnes* biofilm formation by 60%–99% (Kim et al., 2021). Another study also found that six long‐chain PUFAs can inhibit the growth of *Cutibacterium acnes* at 32–1024 mg/L (Desbois & Lawlor, 2013). Research has confirmed that lauric acid effectively reduced ear swelling and granulomatous inflammation caused by *Cutibacterium acnes* in mice with acne through both intradermal injection and epicutaneous application (Nakatsuji et al., 2009). Furthermore, the investigation also discovered that decanoic acid and lauric acid may exert bactericidal and anti‐inflammatory effects on *Cutibacterium acnes* by inhibiting the activation of NF‐κB and the phosphorylation of MAP kinase (Huang et al., 2014).

At present, limited studies have specifically examined the association between the consumption of oily fish and acne. For instance, a previous investigation conducted in Korea revealed that individuals with acne tend to consume a small amount of fish and a significant amount of junk food compared to their healthy counterparts (Jung et al., 2010). Similarly, a study conducted on an Italian population demonstrated that the consumption of fish had a positive effect in terms of reducing the occurrence of moderate and severe acne (Di Landro et al., 2012). Nevertheless, previous research has focused on investigating the relationship between specific components found in oily fish, including fish oil, vitamin D, and PUFAs, and their impact on acne and skin inflammation. These studies have examined the beneficial effects of oral supplementation, topical application, and intravenous injection of fish oil and related active substances such as omega‐3 and omega‐6 PUFAs in promoting skin homeostasis and addressing various dermatological issues (Huang et al., 2018). One study has provided evidence that oily fish, rich in ω‐3 PUFAs, can regulate lipid metabolism and offer protection against acne (Nobili et al., 2016). Additionally, dietary monounsaturated fatty acids (MUFAs) have been shown to improve lipid status due to their anti‐inflammatory properties (Amirkalali et al., 2021). Several studies have also suggested a correlation between levels of EPA and DHA in blood lipids and the consumption of oily fish (Fisk et al., 2018). Combining long‐chain PUFAs with existing treatments for infections caused by *Cutibacterium acnes* and *Staphylococcus aureus* may enhance their therapeutic effectiveness (Desbois & Lawlor, 2013). Furthermore, consuming 300 g/week of oily fish has been found to be feasible and can boost EPA, DHA, and 25 (OH)D levels in children (Vuholm et al., 2020). An investigation conducted through a double‐blind randomized controlled trial demonstrated a noticeable reduction in both inflammatory and non‐inflammatory acne lesions after 10 weeks of supplementation with either omega‐3 fatty acids or γ‐linoleic acid (Jung et al., 2014). Similarly, a large‐scale epidemiological study conducted among teenagers in North Carolina revealed that individuals who consumed substantial quantities of ω‐3 PUFAs, commonly found in fish and seafood, exhibited reduced levels of oily skin as well as fewer occurrences of whiteheads, papules, and pustules (Khayef et al., 2012). Furthermore, a systematic review and meta‐analysis indicated that vitamin D deficiency may be one of the contributing factors in individuals with acne (Wang et al., 2021). Based on these facts, recent research with 257 human beings on a sugar‐restricted diet and a formula using omega 6 and 7, magnesium, and phosphate was able to regress and cure type IV acne (de Souza Pereira, 2023). It is worth noting that this is the first MR study to confirm a strong causal relationship between oily fish intake and acne. However, we do not recommend strictly implementing oily fish supplementation strategies for all acne patients. Instead, individuals who require it should be encouraged to maintain balanced nutrition and consider targeted nutritional supplements to effectively manage skin health.

Emerging evidence suggests a connection between insulin‐like growth factor‐1 (IGF‐1), insulin, androgens, growth hormones, and various growth factors in the regulation of sebaceous gland growth and differentiation (Akpinar Kara, 2022). Higher levels of plasma IGF‐1 have been found to be positively associated with an increased risk and severity of acne (Rahaman et al., 2016). The development and severity of acne may be influenced by conditions such as hyperinsulinemia and insulin resistance (Emiroğlu et al., 2015; Sadowska‐Przytocka et al., 2022). Furthermore, research suggests that consuming foods high in hyperglycemic carbohydrates and insulinotropic milk and dairy products could potentially contribute to acne pathogenesis by promoting insulin growth factor‐1 (IGF‐1) signaling (Melnik, 2015). Moreover, increased insulin levels can contribute to the synthesis of androgens, which in some cases may influence or worsen acne in susceptible individuals (Chen et al., 2002). Scientific research suggests that a low‐fat and low glycemic load may be the cause of the absence of acne among the Kitavan Islanders in Papua New Guinea and the Aché hunters in Paraguay (Cordain et al., 2002). Research has also demonstrated that adhering to a low glycemic index (GI) diet can significantly improve the severity of acne vulgaris and enhance insulin sensitivity in male patients. Interestingly, a significant increase in insulin and IGF‐1 was observed in the group consuming skim milk, but no similar increase was observed in the group consuming meat (Hoppe et al., 2005). On the other hand, acne is a classical dermatosis primarily mediated by androgens, particularly testosterone. The main way in which androgens affect acne is by stimulating sebaceous gland activity and influencing the process of follicular hyperkeratinization (Ju et al., 2017). High insulin levels stimulate the secretion of androgenic hormones and lead to the production of sebum, which is one of the main factors in the development of acne vulgaris (Arora et al., 2011; da Cunha et al., 2013; Moradi Tuchayi et al., 2015; Tasli et al., 2013). Estrogen can help regulate sebum levels by counteracting the stimulation of androgens in the sebaceous glands. Additionally, it possesses anti‐inflammatory properties and can improve clogged pores. So, we hypothesized that fasting insulin, insulin resistance, total testosterone level, and estradiol level might be considered regulated mediators in the relationship between oily fish consumption and acne. In our MR study, we found that consuming a certain amount of oily fish leads to lower fasting insulin levels. However, due to the lack of consideration for exposure factors such as meat quality, genetics, and stress, further research is needed to confirm these results.

To our knowledge, this is the first MR study to explore the causality of 7 types of meat intake on acne. It was also found for the first time that the intake of oily fish could possibly reduce the incidence rate of acne by regulating the fasting insulin level. Our study included a large sample size from diverse populations, ensuring the generalizability of the findings. A comprehensive understanding of the meat related to acne can provide a theoretical foundation for developing targeted and individualized dietary guidelines for acne patients with different types or at different stages of the disease. And it provides ideas for further research on the mechanism underlying the relationship between oily fish and acne.

Our study has some limitations. Firstly, it is challenging to evaluate the generalizability of these results to other populations since all analyses were performed exclusively on European participants. Secondly, due to the unavailability of GWAS data or insufficient instrumental variables in the form of SNPs, we were unable to account for specific nutritional components such as the potential associations between ω‐3 PUFAs, DHA, EPA, and acne. Moreover, we could not differentiate the effects of different dietary combinations and failed to consider factors such as gender, age, family history, stress, and environmental influences. In the future, randomized controlled trials are needed to explore the dose‐effect relationship between oily fish intake and acne. Additionally, it should comprehensively consider the possible confounding factors at an individual level. At the same time, it is necessary to further determine the molecular mechanism of the in vivo action of oily fish components.

## 5 CONCLUSION

Our MR analysis provides evidence that consuming oily fish may reduce the risk of acne. Furthermore, the protective effect of oily fish on acne could be attributed to its ability to decrease fasting insulin levels in the body. However, this study did not identify a causal relationship between acne and the other six types of meat analyzed.

## FUNDING INFORMATION

This research was funded by the National Key Technology R&D Program for the 12th Five‐year Plan of Ministry of Science and Technology, China (No. 2013BAI02B10).

## CONFLICT OF INTEREST STATEMENT

None.

## ETHICS STATEMENT

The GWAS data utilized in this study can be accessed through the IEU Open GWAS project (https://gwas.mrcieu.ac.uk/). Due to the public, anonymized, and de‐identified nature of the data used, this study was exempt from requiring approval from the Ethical Review Authority.

## Supporting information


Figures S1–S11.

Table S1.

Table S2.

Table S3.

Table S4.

## Data Availability

The data can be found in the IEU open GWAS project (https://gwas.mrcieu.ac.uk/). See the supplementary data for details of the instrumental variables estimation finally used in this article.
