# Supplementary material for: Oily fish reduces the risk of acne by lowering fasting insulin levels: A Mendelian randomization study
Source: Food Sci Nutr. 2024 Mar 6;12(6):3964–72. doi: 10.1002/fsn3.4054 (PMC11167188; doi:10.1002/fsn3.4054)
Supplement: Supplementary file 1 — Figures S1–S11. [file FSN3-12-3964-s004.docx]

**
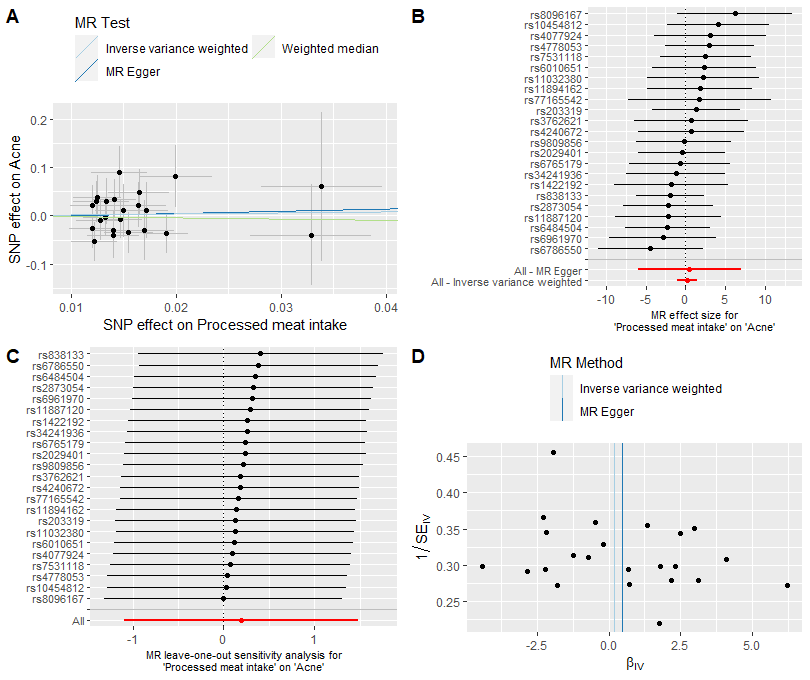
**

**Supplementary Figure 1.** Scatter plot (A), forest plot (B), and “leave-one-out” analysis (C) for MR analysis of processed meat intake and acne, funnel plot (D).


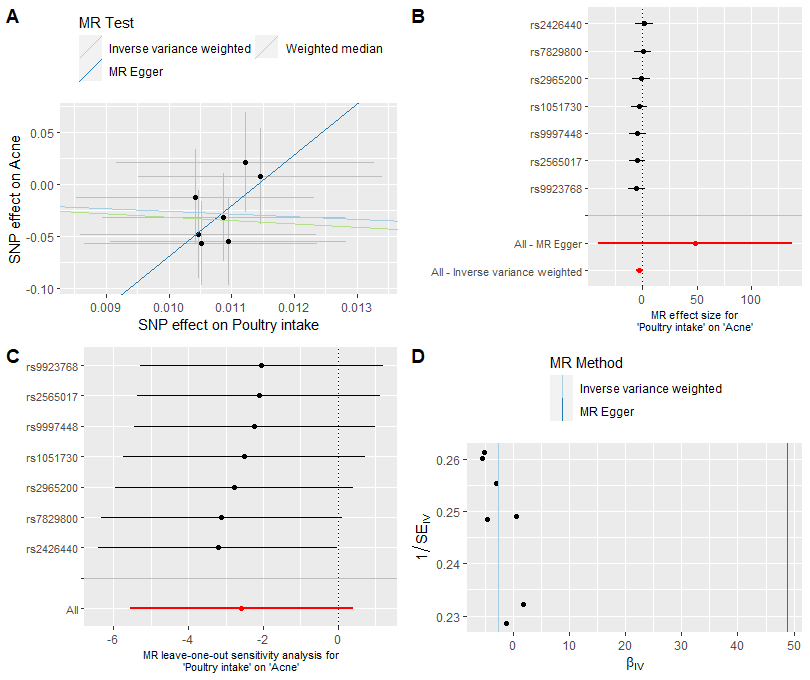


**Supplementary Figure 2.** Scatter plot (A), forest plot (B), and “leave-one-out” analysis (C) for MR analysis of poultry intake and acne, funnel plot (D).


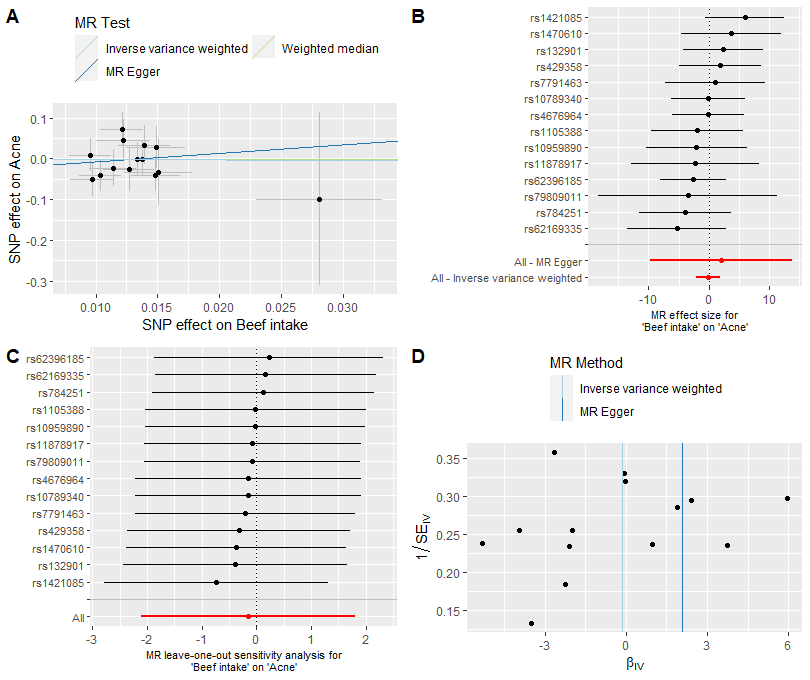


**Supplementary Figure 3.** Scatter plot (A), forest plot (B), and “leave-one-out” analysis (C) for MR analysis of beef intake and acne, funnel plot (D).


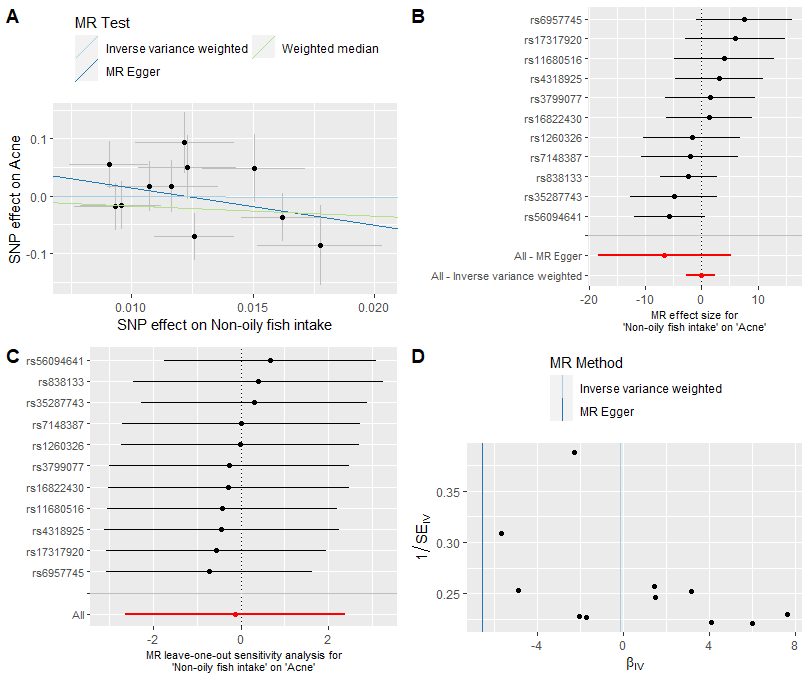


**Supplementary Figure 4.** Scatter plot (A), forest plot (B), and “leave-one-out” analysis (C) for MR analysis of non-oily fish intake and acne, funnel plot (D).


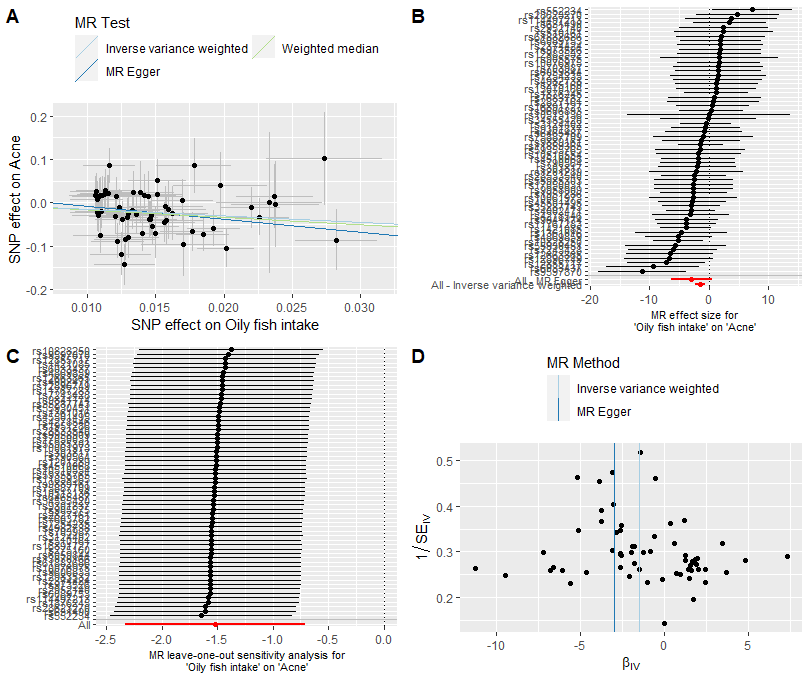


**Supplementary Figure 5.** Scatter plot (A), forest plot (B), and “leave-one-out” analysis (C) for MR analysis of oily fish intake and acne, funnel plot (D).


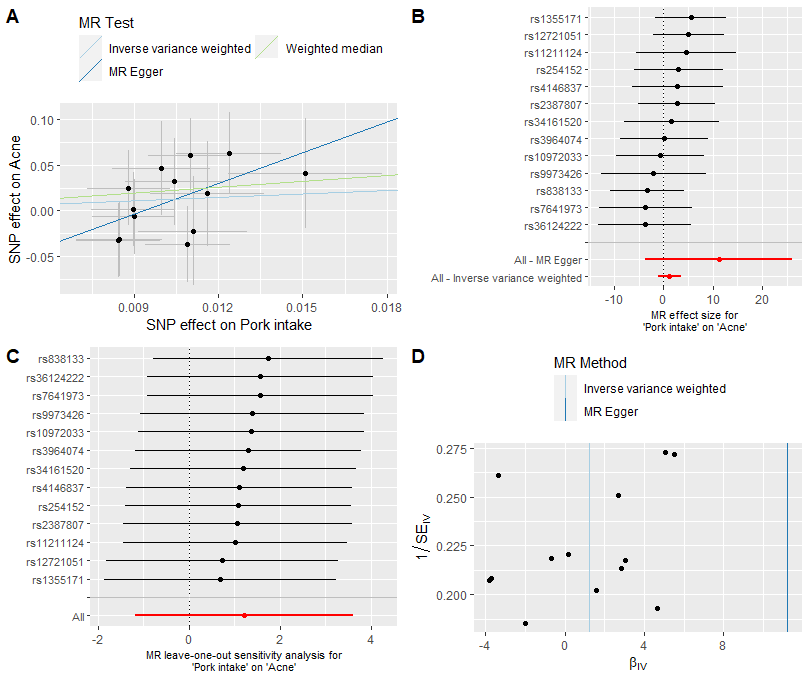


**Supplementary Figure 6.** Scatter plot (A), forest plot (B), and “leave-one-out” analysis (C) for MR analysis of pork intake and acne, funnel plot (D).


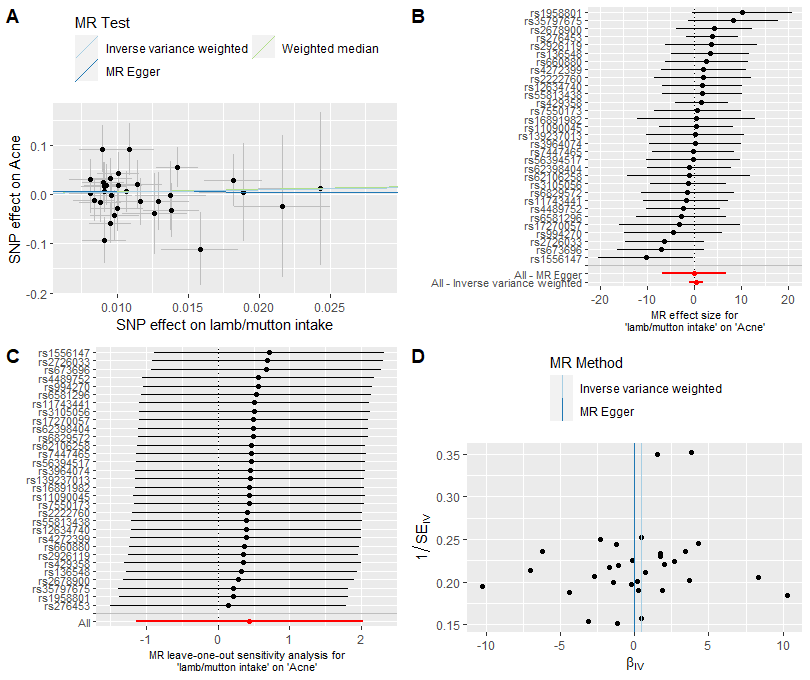


**Supplementary Figure 7.** Scatter plot (A), forest plot (B), and “leave-one-out” analysis (C) for MR analysis of lamb/mutton intake and acne, funnel plot (D).


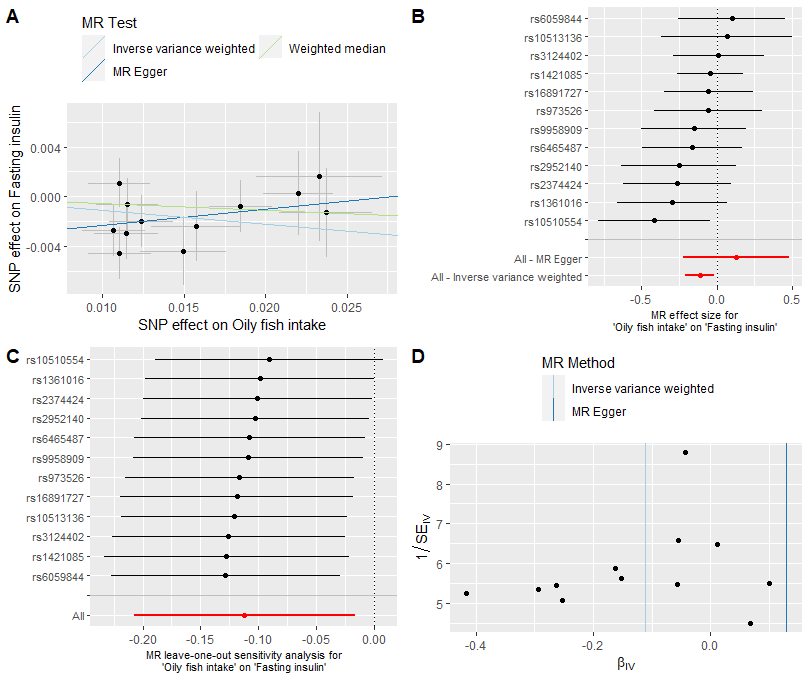


**Supplementary Figure 8.** Scatter plot (A), forest plot (B), and “leave-one-out” analysis (C) for MR analysis of oily fish intake and fasting insulin, funnel plot (D).


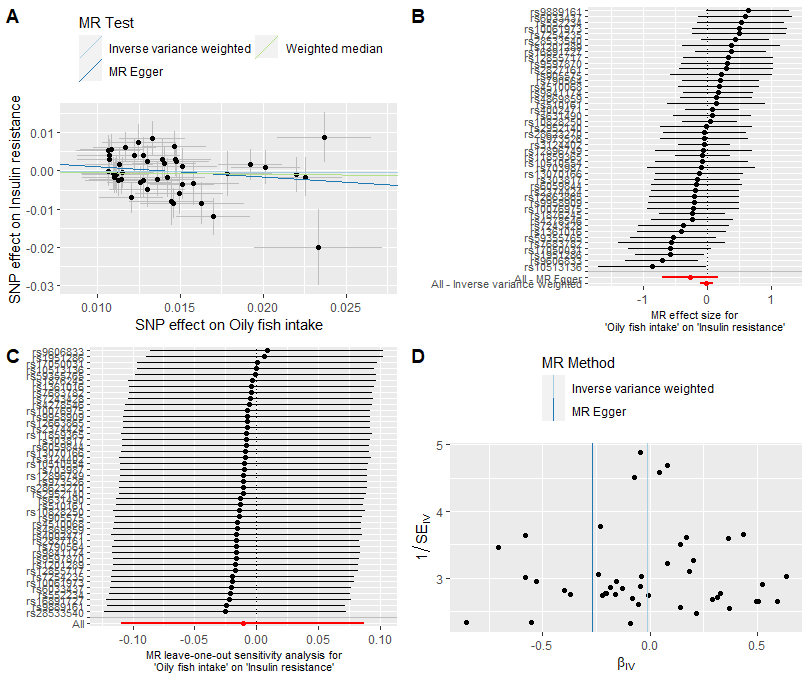


**Supplementary Figure 9.** Scatter plot (A), forest plot (B), and “leave-one-out” analysis (C) for MR analysis of oily fish intake and insulin resistance, funnel plot (D).


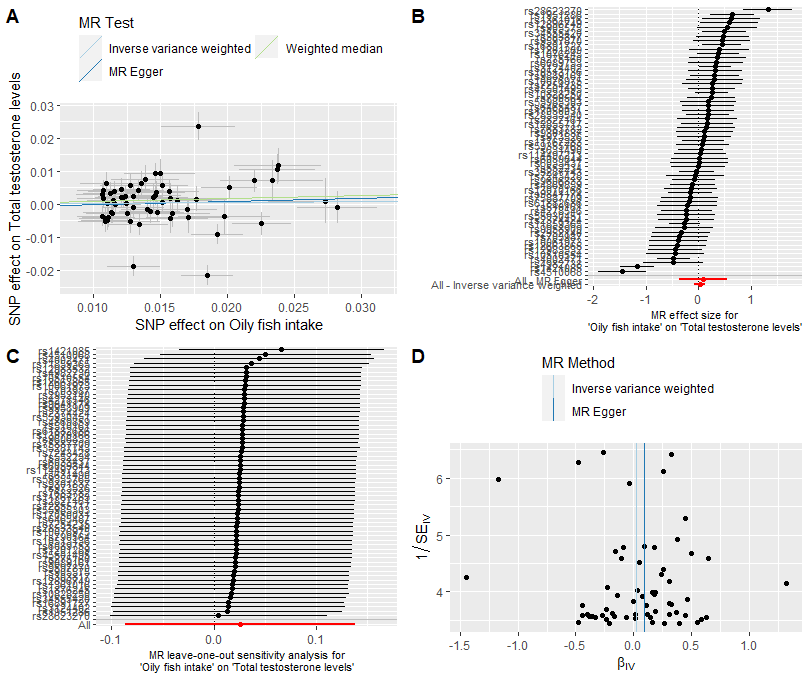


**Supplementary Figure 10.** Scatter plot (A), forest plot (B), and “leave-one-out” analysis (C) for MR analysis of oily fish intake and total testosterone levels, funnel plot (D).


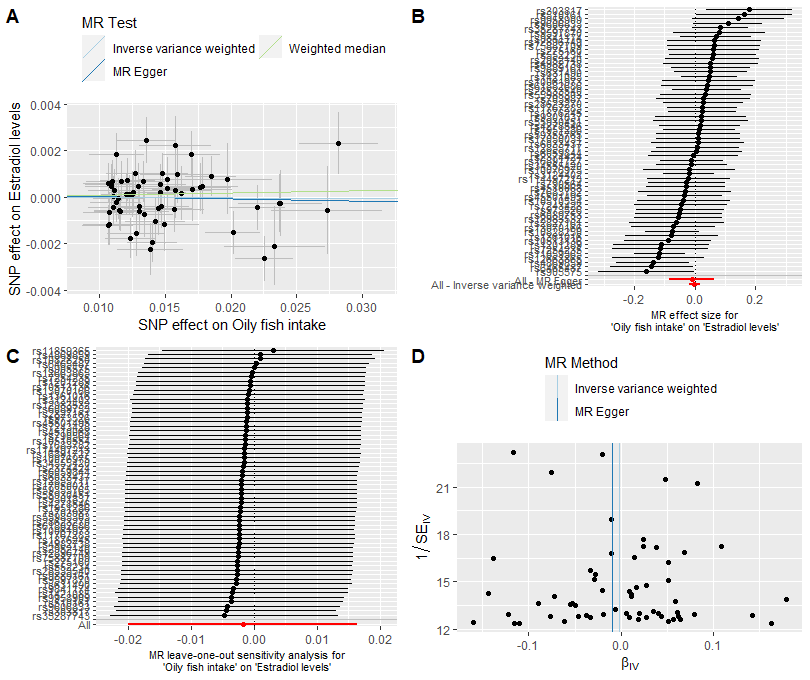


**Supplementary Figure 11.** Scatter plot (A), forest plot (B), and “leave-one-out” analysis (C) for MR analysis of oily fish intake and estradiol levels, funnel plot (D).
